# RNF135 Promoter Methylation Is Associated With Immune Infiltration and Prognosis in Hepatocellular Carcinoma

**DOI:** 10.3389/fonc.2021.752511

**Published:** 2022-01-25

**Authors:** Xiao Wang, Mengke Chen, Xiong Liang, Yu Bai, Judeng Zeng, Xiaoyi Xu, Hao Li, Jing Wang, Keyu Fan, Guijun Zhao

**Affiliations:** ^1^Inner Mongolia Key Laboratory of Endoscopic Digestive Diseases, Endoscopy Center, Inner Mongolia People’s Hospital, Hohhot, China; ^2^Institute of Precision Medicine, The First Affiliated Hospital, Sun Yat-Sen University, Guangzhou, China; ^3^State Key Laboratory of Oncology in South China, Collaborative Innovation Center for Cancer Medicine, Sun Yat-Sen University Cancer Center, Guangzhou, China; ^4^Department of Clinical Medicine, The First Bethune Hospital of Jilin University, Changchun, China; ^5^Anesthesiology, Dalian Medical University, Dalian, China

**Keywords:** Hepatocellular carcinoma, RNF135, DNA methylation, immune infiltration, prognosis

## Abstract

RING finger protein 135 has an important role in the occurrence of many cancers; however its regulation and function of RNF135 in hepatocellular carcinoma remains unknown. The promoter methylation status and mRNA expression of RNF135 was evaluated by methylation-specific PCR, semi-quantitative RT-PCR, and real-time quantitative PCR in HCC tissues and cell lines, and further analyzed from The Cancer Genome Atlas database. Wound healing assay, transwell migration, cell viability, and colony formation assay were performed to investigate the function of RNF135. GSEA analysis, TIMER database, and ESTIMATE algorithm were used to decipher the associated pathway and immune infiltration. The survival analysis was applied to assess the prognostic value of RNF135. RNF135 expression was downregulated in HCC tissues and 5 of 8 HCC cell lines, and was negatively correlated with its promoter hypermethylation. Demethylating regent decitabine restored RNF135 expression on the cellular level. Knockdown of RNF135 expression enhanced the migration of HCC cells, while RNF135 overexpression and decitabine treatment repressed cell migration. Bioinformatics analysis and immunohistochemistry revealed a positive relationship between RNF135 expression and six immune cell infiltrates (B cells, CD4^+^ T cells, CD8^+^ T cells, neutrophils, macrophages, and dendritic cells). Survival analysis disclosed that RNF135 hypermethylation is independently associated with poor clinical outcomes in HCC. Decreased RNF135 expression driven by promoter hypermethylation frequently occurred in HCC and associated with prognosis of HCC. RNF135 functions as a tumor suppressor and is involved in tumor immune microenvironment in HCC.

## Introduction

Liver cancer is the sixth most common cancer worldwide with the fourth highest mortality (1). Hepatocellular carcinoma (HCC) accounts for up to 90% of primary liver cancer (2). Currently, given the difficulty in finding micro-hepatoma in the early-stage (3) and the limited effectiveness of radiotherapy and chemotherapy (4), about 80% of HCC patients are diagnosed at advanced stage and lose the chance of radical surgery, which leads to tumor metastasis and death. Therefore, identifying new biomarkers is urgent and benefits for the individual therapy (5).

The pathogenesis of HCC is complex and multifactorial. Risk factors, such as chronic hepatitis virus infection, alcohol, and aflatoxin, may lead to long-term inflammatory infiltration, metabolic imbalance, and cellular and molecular changes, which cause liver cirrhosis and promote HCC formation and development (6). Over the past few decades, it has become widely accepted that tumor is the result of aberrant activation of oncogenes and inactivation of tumor suppressor genes (7). But more questions about tumor heterogeneity, proliferation, invasion and metastasis, and the development of drug resistance remain unanswered by the heritable variation of genes. Hence, epigenetic modifications are different from classical genetic changes, which regulate gene expression without DNA sequence alteration, could be implicated in cancer (8, 9). DNA methylation is the most widely studied epigenetic modifications compared to histone modification, chromosome remodeling, and non-coding RNA (10).

DNA hypomethylation of proto-oncogenes and overall DNA hypomethylation will increase chromatin instability and leads to tumorigenesis (11, 12). DNA methylation-based biomarkers have been identified for diagnosis and prognosis of HCC (13). Due to reversibility of epigenetics, DNA demethylation drugs such as decitabine, have been applied in clinic and have significant clinical benefits in patients with hematologic tumor. Optimization of drug dosage and mode could harness the potential of demethylation agents to target solid tumors (14, 15).

RING finger protein 135 (RNF135), located on chromosome 17q11.2, belongs to ring finger protein family-containing E3 ubiquitin ligase. The RNF135 protein consists of RING finger domain at N-terminal, and SPRY and PRY motifs at C-terminal. It plays a crucial role in antiviral innate immunity by ubiquitinating RIG-I to produce interferon-β at the early stage of viral infection (16). Liu at el found that RNF135 promoted the proliferation of human glioblastoma cells *via* the ERK pathway (17). In contrast, RNF135 inhibited tumorigenesis *via* regulating AKT signaling in tongue cancer (18). The expression and function of RNF135 in HCC have not been reported.

## Materials and Methods

### Cell Lines and Demethylation Treatment

Human HCC cell lines (Bel-7402, HepG2, Huh7, MHCC-97H, MHCC-97L, SMMC-7721, SNU-387, and SNU-449) and immortalized normal liver cell line LO2 were purchased from American Type Culture Collection and Cell Resource Center of Shanghai Institutes. HepG2, Huh7, MHCC-97H, MHCC-97L, and LO2 were cultured in DMEM medium (Gibco) supplemented with 10% FBS (BI). Bel-7402, SMMC-7721, SNU-387, and SNU-449 were cultured in RPMI1640 medium (Gibco) supplemented with 10% FBS (BI). For demethylation treatment, HCC cells were treated with 10μM decitabine (Selleck, S1200) for 3 days and harvested for the following experiments.

### Clinical Samples

A total of 23 primary tumors and paired adjacent non-tumors tissues were collected from HCC patients diagnosed by at least two pathologists from the Sun Yat-Sen University Cancer Center (SYSUCC). These patients did not receive any therapeutic intervention and have written informed consents before surgery. This study was approved by the Ethics Committee of SYSUCC.

### Bisulfite Conversion and Methylation-Specific PCR

Genomic DNA from HCC cell lines and tissues was isolated using the TIAN-amp Genomic DNA Kit (TIANGEN BIOTECH, DP304). Genomic DNA was bisulfite-modified using the EZ DNA Methylation-Gold Kit (Zymo, D5006). Two primer pairs were designed to identify methylated and unmethylated sites by performing methylation-specific PCR (MS-PCR) to amplify the bisulfite-modified DNA. The methylation-specific PCR forward and reverse primer sequences were 5’-ATTCGTTCGGTTTAATTTCGAC-3’ and 5’-CAAAACCTCCAAACAATAACGAC-3’, respectively. The non-methylation-specific PCR forward and reverse primer sequences were 5’-GGAGATTTGTTTGGTTTAATTTTGAT-3’ and 5’-ACAAAACCTCCAAACAATAACAAC-3’, respectively.

### Semi-Quantitative RT-PCR and Real-Time Quantitative PCR

Trizol reagent (Ambion, 15596018) was used to isolate total RNA. Go Script Reverse Transcription Kit (Promega, USA, A5001) was used to synthesize cDNA. Semi-quantitative RT-PCR and real-time quantitative PCR (qPCR) were performed to detect the expression of RNF135. Reactions were performed in triplicate, and GAPDH was used as an internal control. The primers were listed as follow: RNF135-RT Forward, 5’-TACTGGGAAGTGGACACTAGGAATT-3’,

RNF135-RT Reverse, 5’-CTTGACCATGTGCCATGCA-3’, GAPDH- RT Forward, 5’-CTCCTCCTGTTCGACAGTCAGC-3’, and GAPDH-RT Reverse, 5’-CCCAATACGACCAAATCCGTT-3’.

### Knockdown and Overexpression RNF135 in Cell Lines

Small interference RNA (si-1: 5’-GAGAGACUCUGCAAGCUAU-3’, si-2: 5’-GGAUCGUAGUGAAACCGAU-3’) and control sequence (5’-GTTCTCCGAACGTGTCAC-3’) were purchased from Shanghai Gene Pharma. RNF135 overexpression plasmid was generated by cloning RNF135 cDNA into pcDNA3.1 vector. They were transfected into cells using Lipofectamine 3000 (Invitrogen, USA) according to the manufacturer’s protocol.

### Western Blotting

Total protein was extracted using RIPA Lysis Buffer (Beyotime, P0013B) with PMSF (Beyotime, ST506). Protein concentration was then measured by BCA protein assay kit (Thermo Fisher). Sample proteins (20 μg) were separated by sodium dodecyl sulfate polyacrylamide gel electrophoresis (SDS-PAGE) on 10% gel and blotted onto the polyvinylidene difluoride (PVDF) membrane. The membrane was blocked with 5% milk for 1 h at room temperature (RT), then incubated with primary antibody at 4°C overnight. Next, the secondary antibody incubation was applied at RT for 1 h. The blot signals were visualized by enhanced chemiluminescence. Anti-RNF135 (Invitrogen, PA554457, 1:1,000) and anti-GAPDH (CST5174, 1:5,000) antibodies were used as primary antibodies.

### Transwell Migration Assay

Suspended in serum-free medium were 5 × 10^4^ cells which were seeded onto the upper chamber (Corning), and lower chamber contained 700 μl of medium with 10% FBS. The chambers were incubated at 37°C with 5% CO_2_ for 10 h. After wiping the upper chamber with cotton swabs, the chamber was fixed with 4% paraformaldehyde and stained with crystal violet.

### Wound Healing Assay

Confluent cell monolayers were scratched with 100 μl pipette tip vertically and horizontally to create a wound area in all experimental groups, washed with medium and photographed to record the initial wound width. Cells were cultured in serum-free medium for 24 h and imaged the wound to determine the residual wound width. The wound closure (%) = (initial wound width − residual wound width)/initial wound width × 100%.

### Colony Formation Assay and Cell Viability

Following transfected with siRNA or overexpressing plasmid for 2 days, cells were seeded in 6-well plate with 800 cells in each well and cultured in a CO_2_-incubator at 37°C for 2 weeks. Cells were stained with crystal violet and counted. Approximately 1,000 cells/well were seeded in 96-well plate after transfected with siRNA or overexpressing plasmid for 2 days. Cell viability was detected at regular intervals by Cell counting kit (Dojindo) following the manufacturer’s protocol. After incubating with 10 μl regent for 2 h, the absorbance at 450 nm was detected by a microplate reader. Also, HCC cells were treated with decitabine for 3 days and followed experiments.

### Immunohistochemistry

Immunohistochemistry was performed on paraffin sections of normal and HCC tissues using anti-RNF135 antibody, anti-CD20 antibody, anti-CD8 antibody, anti-CD4 antibody, anti-CD68 antibody, anti-CD15 antibody, and anti-141 antibody. Protein expression in the immunohistochemical staining was quantified according to the immunohistochemical score. The final score was analyzed by staining intensity score and the percentage score.

### Gene Enrichment Analysis

We used Rstudio software to develop a program to simulate the high or low expression of RNF135 by selecting 30 samples randomly that had the 25% highest or 25% lowest RNF135 expression values and then used this gene set for GSEA.

### Estimation of Stromal and Immune Cells in Malignant Tumor Tissues Using Expression Data (ESTIMATE)

Tumor purity and the infiltration of stromal/immune cells were predicted with ESTIMATE algorithm applied to the expression data downloaded from The Cancer Genome Atlas (TCGA) (19). The R packages estimate, limma, ggplot2, ggpubr, and ggExtra were used for graphical visualization of the results.

### Databases and Statistical Analysis

Gene expression, DNA methylation, and clinical information data from the TCGA were downloaded from UCSC Xena (https://xenabrowser.net/) and all datasets above were from the same cohort. GraphPad Prism 7.0 and IBM SPSS 26.0 were applied for plotting and statistical analysis. Student’s t-test for unpaired, Spearman’s correlation, Kaplan–Meier survival analysis, and log-rank test, and Cox regression model were used in this study. A p-value less than 0.05 is considered statistically significant.

## Results

### RNF135 Expression is Downregulated in HCC Tissues

**Figure 1:**
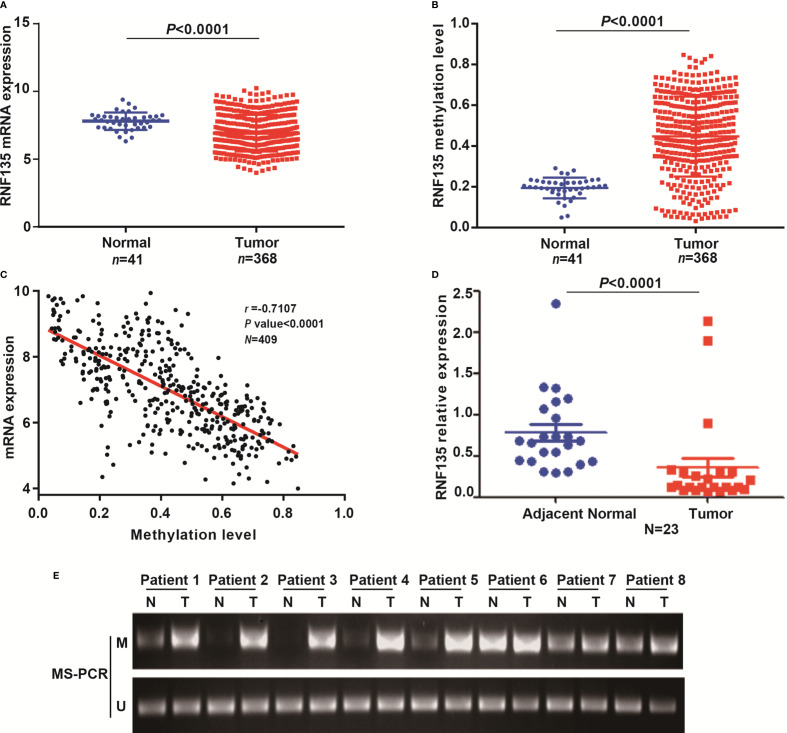
RNF135 expression and methylation levels and analysis of their correlation in HCC tissues. **(A)** RNF135 mRNA expression data from TCGA were analyzed. **(B)** The average methylation level of 10 CpG sites was used to evaluate promoter methylation of RNF135. Promoter methylation levels in HCC tissues and adjacent non-tumor tissues were analyzed with TCGA data. **(C)** The correlation between RNF135 mRNA expression and methylation status was analyzed with TCGA data (Spearman’s correlation r = −0.7017, n = 409, *p*-value <0.0001) **(D)** RNF135 mRNA expression was measured by real-time quantitative PCR in 23 pairs of samples from SYSUCC. **(E)** MS-PCR (M, methylation; U, unmethylation) was used to detect methylation status in 8 pairs of samples from SYSUCC.

To verify the association between RNF135 expression and methylation in HCC tissues, we analyzed data from the TCGA. The methylation level at promoter was evaluated by the average methylation level of 10 CpG sites around transcription start sites (**Supplementary Figure 1**). RNF135 expression was significantly downregulated (**Figure 1A**) and hyper-methylated (**Figure 1B**) in HCC tissues compared with adjacent non-tumor tissues. Furthermore, there was a significant negative correlation between expression and methylation level (*r* = −0.7107, *P <*0.0001, **Figure 1C**). Correlation analysis between RNF135 methylation status and clinical characteristics in HCC from the TCGA revealed that RNF135 methylation status was significantly related to HBV/HCV infection (*P* = 0.011) and recurrence/progression (*P* = 0.018) (**Table 1**). To validate these results from the TCGA data, we detected the mRNA expression level of RNF135 in 23 pairs of HCC tissue samples, and 8 randomly selected samples were analyzed by MS-PCR. The studies provided consistent evidence that RNF135 was lowly expressed (**Figure 1D**) and hyper-methylated in most HCC tissues (**Figure 1E**). Hence, RNF135 was downregulated in HCC tissues on account of the promoter hypermethylation. These results established that RNF135 may act as a potential tumor suppressor in HCC.

**Table 1:** The association between RNF135 promoter methylation level and clinical characteristics of patients with HCC from the TCGA.

	Low methylation		High methylation		
Variable	n	%	n	%	P-value
**Sex**					
Male	129	52.40%	117	47.60%	0.157
Female	53	44.50%	66	55.50%	
**Age**					
<60	85	49.10%	88	50.90%	0.791
≥60	97	50.50%	95	49.50%	
**Family history of cancer**					
No	109	47.20%	122	52.80%	0.051
Yes	72	58.10%	52	41.90%	
**HBV/HCV infection**					
No	115	55.80%	91	44.20%	**0.011**
Yes	66	42.30%	90	57.70%	
**Alcohol consumption**					
No	117	48.50%	124	51.50%	0.435
Yes	64	52.90%	57	47.10%	
**Tumor stage**					
I & II	134	49.30%	138	50.70%	0.832
III & IV	46	50.50%	45	49.50%	
**Vascular invasion**					
No	123	51.50%	116	48.50%	0.281
Yes	55	45.50%	66	54.50%	
**Recurrence/Progression**					
No	64	43.20%	84	56.80%	**0.018 **
Yes	94	56.60%	72	43.40%	

Bold values represent significant differences.

### RNF135 is Epigenetically Downregulated by Promoter Methylation in HCC Cell Lines

**Figure 2:**
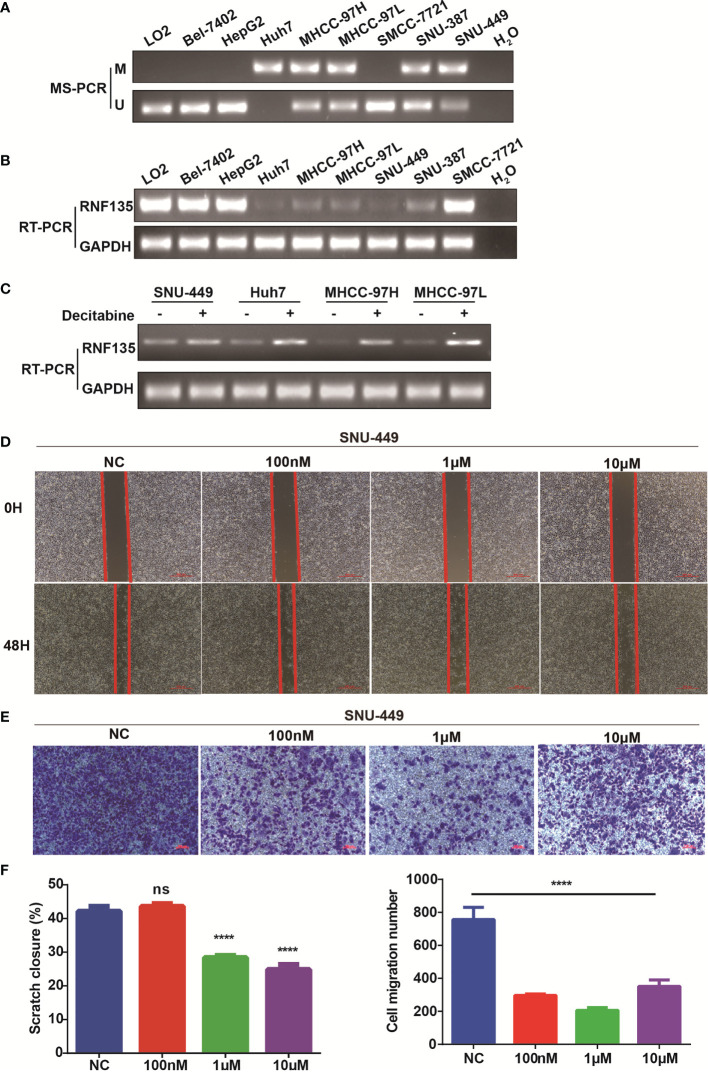
Correlation between RNF135 methylation and mRNA expression in HCC cell lines. **(A)** The methylation status in immortalized normal liver cell line LO2 and HCC cell lines (Bel-7402, HepG2, Huh7, MHCC-97H, MHCC-97L, SMMC-7721, SNU-387, and SNU-449) were measured by MS-PCR (M, methylation; U, unmethylation). H_2_O was used as a negative control. **(B)** The expression levels in cell lines were determined by semi-quantitative RT-PCR, and H_2_O was used as a negative control. **(C)** RNF135 mRNA expression was partially restored after treatment with the demethylation reagent decitabine in 4 HCC cell lines whose promoter region was hypermethylated. **(D–F)** Wound healing assay and transwell assay were used to detect cell migration and invasion (up), and one-way ANOVA was applied between experimental group and control group (down). ****P < 0.0001, ns represents no significance.

The methylation status of RNF135 promoter in HCC cell lines were analyzed by MS-PCR. Compared with normal liver cell line LO2, the promoter region of RNF135 was hypermethylated in 5 of 8 HCC cell lines (**Figure 2A**) and its expression was low in these five cell lines (**Figure 2B**). Decitabine treatment restored RNF135 expression in 4 hypermethylated HCC cell lines (SNU-449, Huh7, MHCC-97H, MHCC-97L) (**Figure 2C**). These results showed RNF135 expression was downregulated partially due to promoter hypermethylation in HCC cell lines, and demethylation agent could rescue its expression. Further, the wound healing assay showed decitabine treatment dramatically suppressed the migration distance of cells and the transwell assay confirmed fewer invasive cells after decitabine treatment (**Figures 2D–F**).

### Loss of RNF135 Promotes Cell Migration But Not Cell Proliferation in HCC

**Figure 3:**
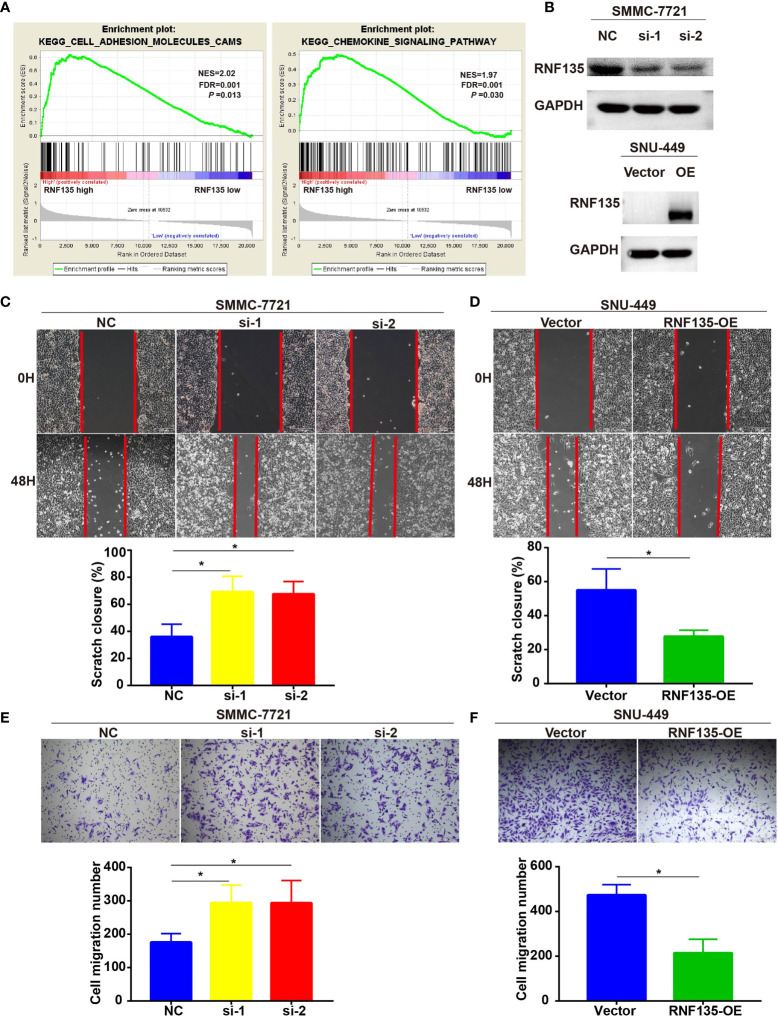
The effects of RNF135 expression on cell migration and invasion. **(A)** RNF135 related pathways in HCC patients were analyzed by GSEA. **(B)** Knockdown and overexpression efficiency of RNF135 in cell lines was measured by western blot. **(C, D)** Wound healing assay was used to detect cell migration (up), and Student’s t-test for unpaired was applied between experimental group and control group (down). **(E, F)** Transwell assay was used to evaluate cell invasion (up), and Student’s t-test for unpaired was applied between experimental group and control group (down). All experiments were repeated three times, and data were expressed as mean ± SD. * P < 0.05.

To characterize the function of RNF135 as a tumor suppressor in HCC, we performed GSEA analysis using the TCGA data. We found differentially expressed genes were enriched in cell adhesion molecules (CAMs) (NES = 2.02, FDR = 0.001 *P*-value = 0.013) and chemokine signaling pathways (NES = 1.97, FDR = 0.001, *P*-value = 0.030) (**Figure 3A**). CAMs are often found involved in migration (20) and chemokines and chemokine receptors contributes to cancer metastasis (21). Therefore, we assessed the effect of RNF135 on cell migration by knocking down RNF135 in SMCC-7721 (high endogenous RNF135 expression) and overexpressing in SNU449 (low endogenous RNF135 expression) (**Figure 3B**). The wound healing assay showed loss of RNF135 in HCC cells dramatically enhanced the migration distance of cells, whereas overexpression of RNF135 suppressed the wound healing capability (**Figures 3C, D****)**. The transwell assay further confirmed that there were more invasive cells after knocking down RNF135, but fewer invasive cells after RNF135 overexpression (**Figures 3E, F****)**. We conducted colony formation and cell viability assay to evaluate the effect of RNF135 on cell growth and proliferation, and found that neither knockdown nor overexpression of RNF135 affected cell proliferation and clonal formation. Taken together, these results proved that RNF135 expression significantly inhibited cell migration of HCC cells and RNF135 downregulation increased HCC cell migration.

### RNF135 is Related to Immune Infiltration

**Figure 4:**
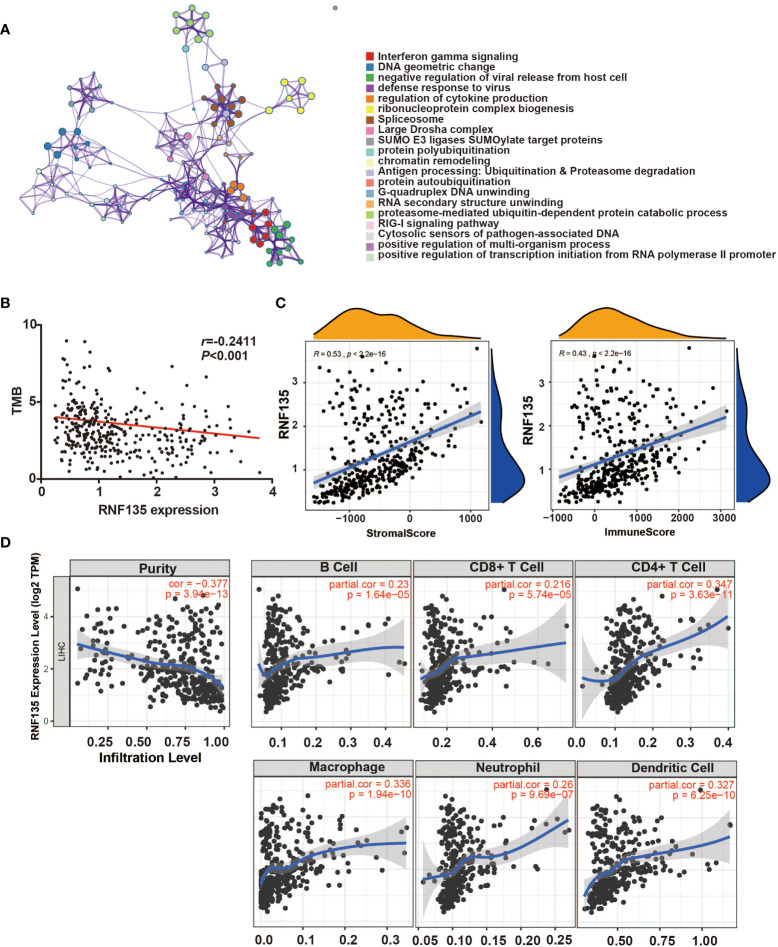
RNF135 expression associations with immune infiltration in HCC tissues. **(A)** The enrichment of functions and signaling pathways of the target genes was conducted by Metascape. **(B)** The correlation between RNF135 expression and TMB (Spearman’s correlation r = −0.2411, *p*-value <0.001). **(C)** ESTIMATE was used to predict the association between RNF135 expression with the presence of infiltrating stromal/immune cells in tumor tissues: stromal score (quantifies the presence of stroma in tumor tissue); immune score (that represents the infiltration of immune cells in tumor tissue). **(D)** The associations between RNF135 expression with tumor purity and six immune cell types (B cell, CD8^+^ T cell, CD4^+^ T cell, macrophage, neutrophil, and dendritic cell) in the tumor microenvironment were estimated by TIMER.

**Figure 5:**
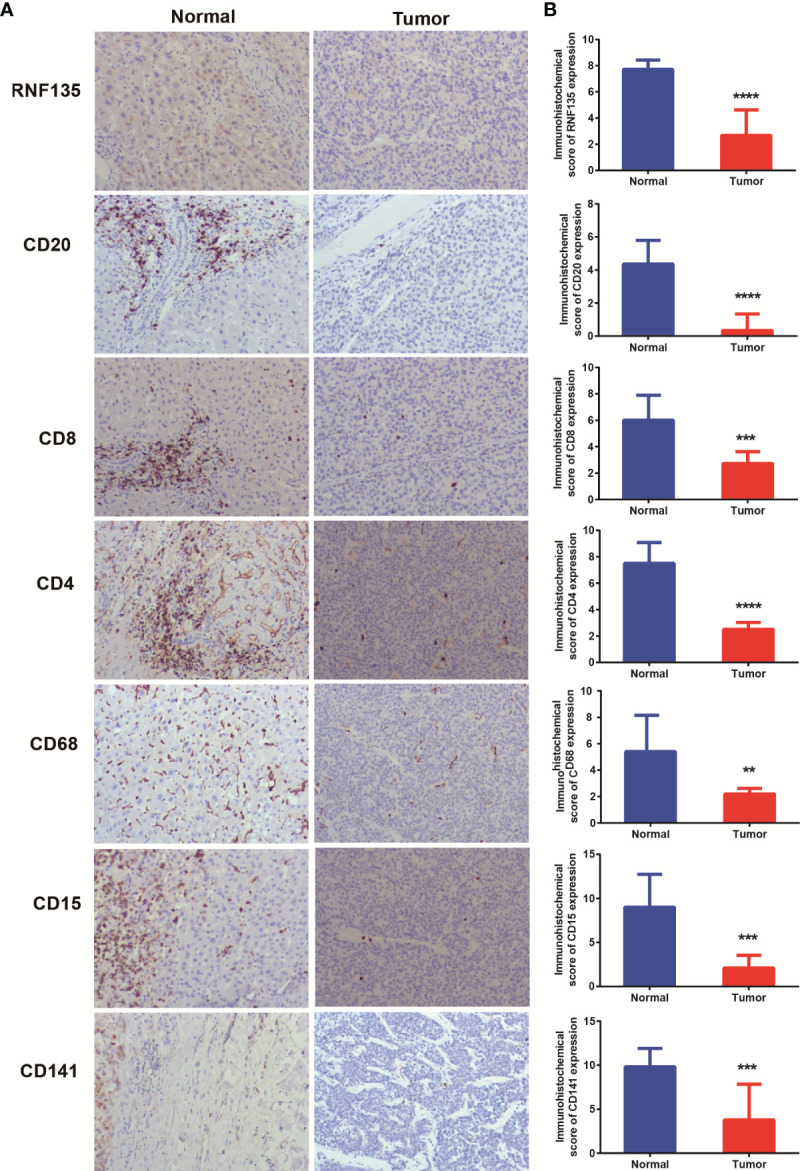
Immunohistochemistry detected the expression of immune cells in HCC samples **(A)** Expression of RNF135, CD20, CD8, CD4, CD68, CD15, and CD141 in normal and HCC tissues. **(B)** Immunohistochemical scores of RNF135, CD20, CD8, CD4, CD68, CD15, and CD141 in normal and HCC tissues. **P ≤ 0.01, ***P ≤ 0.001, ****P ≤ 0.0001.

A total of 258 potential substrates of RNF135 from UbiBrowser (http://ubibrowser.ncpsb.org/ubibrowser/) were analyzed by Metascape (https://metascape.org). Pathways included negative regulation of viral release from host cell and defense response to virus were recognized (**Figure 4A**), which is consistent with the role of RNF135 in antiviral innate immunity (16). In addition, other immune system pathways, such as interferon gamma signaling and regulation of cytokine production was also found (**Figure 4A**), suggesting that RNF135 may have potential effects on anti-tumor immune and immune therapy. The negative correlation between RNF135 expression and tumor mutational burden (TMB) reinforced our suspicion (*r* = −0.2411, *p <*0.001) (**Figure 4B**). High TMB was considered as a consequence of exposure to carcinogens, leading to the production of neoantigens, which was widely used a predictable biomarker for the response to immune checkpoint blockade (22, 23). Additionally, RNF135 was positively correlated with stromal score and immune score (**Figure 4C**). The correlation between RNF135 and different types of immune cells were obtained from TIMER database (https://cistrome.shinyapps.io/timer/). From the results, we recognized the negative correlation between RNF135 expression and tumor purity (Cox = 0.377, *p* = 3.94e−13) and the positive correlation between RNF135 expression and the infiltration of immune cells, including B cells (Cox = 0.23, *p* = 1.64e−05), CD8^+^ T cell (Cox = 0.216, *p* = 5.74e−05), CD4^+^ T cell (Cox = 0.347, *p* = 3.63e−11), macrophage (Cox = 0.336, *p* = 1.94e−10), neutrophil (Cox = 0.26, *p* = 9.69e−07), and dendritic cell (Cox = 0.327, *p* = 6.25e−10) (**Figure 4D**). Immunohistochemistry showed RNF135 expression and six immune cell types (B cell, CD8^+^ T cell, CD4^+^ T cell, macrophage, neutrophil, and dendritic cell) were high in normal HCC tissues and the expression were low in tumor tissues (**Figures 5A, B**).

### Methylation Status of RNF135 is an Independent Predictor of HCC Patients’ Prognosis

**Figure 6:**
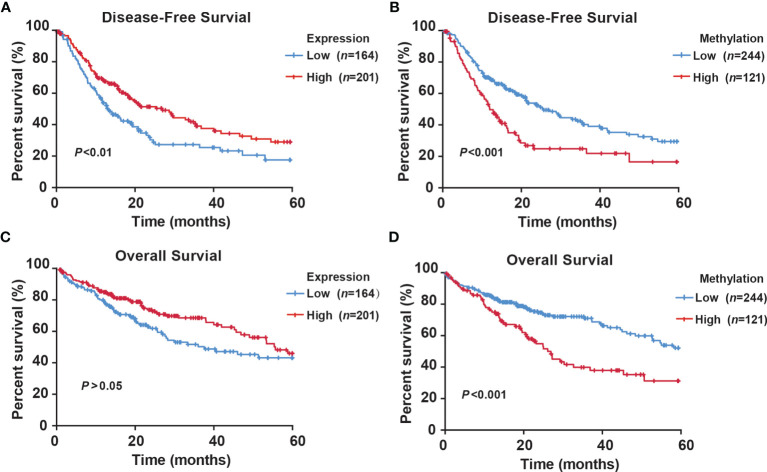
Kaplan–Meier survival curve comparing high and low levels of RNF135 expression or methylation in HCC patients from the TCGA. **(A)** The disease-free survival curve for HCC patients with high or low expression level of RNF135. **(B)** The disease-free survival curve for HCC patients with high or low methylation level of RNF135. **(C)** The overall survival curve for HCC patients with high or low expression level of RNF135. **(D)** The overall survival curve for HCC patients with high or low methylation level of RNF135.

To explore the prognostic value of RNF135 expression and methylation, we analyzed data of HCC patients from TCGA. The clinical features of HCC patients linked to the survival status are shown in **Table S1**. Tumor stage was a significant prognostic factor (*P <*0.001). Moreover, RNF135 methylation (*P* = 0.001), family history of cancer (*P* = 0.038), and HBV/HCV infection (*P* = 0.003) were found to be related to death. Using univariate Cox regression analysis, RNF135 methylation (HR = 1.960; 95% confidence interval (CI): 1.367–2.810; *P <*0.001), tumor stage (HR = 2.375; 95% CI: 1.646–3.426; *P <*0.001) were associated with increased risk of HCC death. Unexpectedly, HBV/HCV infection decreased the mortality risk (HR = 0.541; 95% CI: 0.368–0.795; *P* = 0.002) (**Table S2**). Subsequently, multivariate Cox regression analysis showed that high RNF135 methylation predicted poorer survival of HCC patients (HR = 1.912; 95% CI: 1.322–2.766; *P* = 0.001) (**Table 2**). As shown in the Kaplan–Meier survival curves, patients with high RNF135 expression (median disease-free survival time (DFS) = 32.850 months) had a significantly longer DFS than those with lower expression (median DFS = 25.349 months) (**Figure 6A**). Low methylation was associated with better DFS in HCC patients (median DFS: 32.857 vs 22.860 months) (**Figure 6B**). There was also a trend for longer survival in patients with higher RNF135 mRNA level (median overall survival time (OS): 43.926 vs 37.845 months) and low methylation (median OS: 44.959 vs 33.668 months) (**Figures 6C, D**). These data suggested that RNF135 hypermethylation was an independent predictor for the poor prognosis of HCC patients.

**Table 2:** Multivariate Cox regression analysis of potential independent prognostic factors for HCC patients.

Variable	HR (95% CI)	*P*-value
**Sex**		
Male	1.000	0.511
Female	0.876 (0.590–1.300)	
**Age**		
<60	1.000	0.507
≥60	1.136 (0.779–1.657)	
**HBV/HCV infection**		
No	1.000	**0.021**
Yes	0.619 (0.412–0.93)	
**Tumor stage**		
I & II	1.000	**0.001**
III & IV	1.951 (1.323–2.887)	
**Methylation**		
Low	1.000	**0.001**
High	1.912 (1.322–2.766)	

Bold values represent significant differences.

## Discussion

In the present study, we confirmed RNF135 was frequently silenced in HCC tissues and cell lines due to promoter hypermethylation using MS-PCR and RT-PCR. RNF135 expression in cell lines was restored with demethylating reagent, which proved promoter methylation tend to relate negatively to its expression. It was in agreement with this phenomenon of methylation-dependent gene silencing (24, 25). Our functional experiments demonstrated that RNF135 expression could strikingly inhibit HCC cells migration *in vitro*, which explained the results of GSEA using HCC expression data from the TCGA. It has been reported that cell-extracellular matrix adhesion and cell–cell adhesion related to tumor cell metastasis (26). Chemokines interact with their receptors to direct cells to specific location, affecting the tumor microenvironment and survival of malignant cell (27). In conclusion, RNF135 may act as a tumor suppressor, regulating HCC cell migration.

It is well established that RNF135 is involved in antiviral immunity process. During viral infection, RNF135 activate RIG-I receptor signaling to promote the release of interferon-β (16, 28). Hepatitis virus infection is the major causative factor for HCC (29). Our analysis found that hepatitis virus infection in patients is a protective factor for prognosis. RNF135 expression and methylation level was associated with hepatitis virus. These findings were consistent with the theory that antiviral immunity could enhance the anti-tumor effect (30, 31). When patients were infected by hepatitis virus, RNF135 expression might be upregulated by decreasing methylation to enhance antiviral immunity. Such tumor environment is beneficial for immune cells killing tumor cells. More importantly, we found that RNF135 was positively correlated with immune infiltration. Since RNF135 was positively correlated with all six types of immune cells, it is undeniable that RNF135 is related to anti-tumor immunity.

Previous studies have presented that the combination of DNA demethylation drugs and immunotherapy may be a potential therapeutic strategy. Recently, Roulois et al. revealed that low-dose decitabine treatment induced the expression of interferon-stimulated genes through activating MDA5/MAVS/IRF7 pathway in colon cancer cells (31). Similarly, in ovarian cancer cells, 5-azacytidine and 5-Aza-CdR could trigger cytosolic dsRNA-sensing and upregulate type I interferon-response genes expression, reported by Chiappinelli et al. (30). Some studies proposed that immune genes could be directly regulated by DNA methylation. Liu et al. found a crosstalk between DNA methyltransferase 1 and PD-L1 in sorafenib resistant HCC cells (32). A phase II clinical trial in acute myeloid leukemia revealed demethylation agents, like azacytidine, could upregulate mRNA expression of PD-L1, PD-L2, PD-1, and CTLA-4 (33). Understanding how DNA methylation interacts with immune system might provide insight into expanding the range of patients suitable for immunotherapy. In our study, RNF135 was proposed to be the link between DNA methylation and immunity and had a potential to serve as biomarker for immunotherapy or therapeutic target.

Since DNA methylation regulates RNF135 expression, demethylation drugs may enhance immune infiltration by increasing the expression of RNF135. Immune-hot tumors response to immunotherapy well (34). High TMB is an emerging biomarker for immunotherapy (35). The expression of RNF135 was negatively correlated with TMB, which hinted there are some patients with low RNF135 expression, low immune infiltration but high TMB. It may reduce the accuracy of TMB in predicting response of the patient to immunotherapy. Therefore, combining TMB and RNF135 expression may be a useful biomarker in HCC patient selection for immune checkpoint inhibitor. However, our findings are based on only bioinformatics analysis, and further experimental verification is needed. Based on the relationship with immune infiltration and the function of RNF135 in regulating cell migration, we identified RNF135 as a prognostic factor for clinical application. Low methylation of RNF135 promoter (responsible for high expression) predicted better disease-free survival and overall survival in HCC patients.

In conclusion, we demonstrated that RNF135 was epigenetically silenced in HCC and served as a tumor suppressor in HCC development. Hypermethylation of RNF135 was associated with poor survival in HCC patients. RNF135 may serve as a bridge between DNA methylation and immune system to establish precision medicine for guiding drug combination.

## Data Availability Statement

The original contributions presented in the study are included in the article/**Supplementary Material**. Further inquiries can be directed to the corresponding author.

## Ethics Statement

The studies involving human participants were reviewed and approved by the Ethics Committee of SYSUCC. The patients/participants provided their written informed consent to participate in this study.

## Author Contributions

XW supplemented the experiments and revised the manuscript. MC performed experiments, analyzed data, and drafted the manuscript. XL conceived the study and YB participated in analyzing data. JZ, XX, and HL performed experiments and interpreted results. JW and KF participated in the literature search. GZ provided financial support and overall supervision, analyzed and interpreted the data. All authors contributed to the article and approved the submitted version.

## Funding

This work was supported by grants from the National Natural Science Foundation of China (81960539), the Science and Technology Project of Inner Mongolia autonomous region (2019GG114), and the Natural Science Foundation of Inner Mongolia (2018MS08007).

## Conflict of Interest

The authors declare that the research was conducted in the absence of any commercial or financial relationships that could be construed as a potential conflict of interest.

## Publisher’s Note

All claims expressed in this article are solely those of the authors and do not necessarily represent those of their affiliated organizations, or those of the publisher, the editors and the reviewers. Any product that may be evaluated in this article, or claim that may be made by its manufacturer, is not guaranteed or endorsed by the publisher.

## References

[1] Bray F Ferlay J Soerjomataram I Siegel RL Torre LA Jemal A. Global Cancer Statistics 2018: GLOBOCAN Estimates of Incidence and Mortality Worldwide for 36 Cancers in 185 Countries. CA: Cancer J Clin (2018) 68(6):394–424. doi: 10.3322/caac.21492 30207593

[2] Sayiner M Golabi P Younossi ZM. Disease Burden of Hepatocellular Carcinoma: A Global Perspective. Dig Dis Sci (2019) 64(4):910–7. doi: 10.1007/s10620-019-05537-2 30835028

[3] El-Serag HB. Hepatocellular Carcinoma. New Engl J Med (2011) 365(12):1118–27. doi: 10.1056/NEJMra1001683 21992124

[4] Forner A Reig M Bruix J. Hepatocellular Carcinoma. Lancet (2018) 391(10127):1301–14. doi: 10.1016/s0140-6736(18)30010-2 29307467

[5] Schulze K Nault JC Villanueva A. Genetic Profiling of Hepatocellular Carcinoma Using Next-Generation Sequencing. J Hepatol (2016) 65(5):1031–42. doi: 10.1016/j.jhep.2016.05.035 27262756

[6] Villanueva A. Hepatocellular Carcinoma. New Engl J Med (2019) 380(15):1450–62. doi: 10.1056/NEJMra1713263 30970190

[7] Vogelstein B Kinzler KW. Cancer Genes and the Pathways They Control. Nat Med (2004) 10(8):789–99. doi: 10.1038/nm1087 15286780

[8] Feinberg AP Tycko B. The History of Cancer Epigenetics. Nat Rev Cancer (2004) 4(2):143–53. doi: 10.1038/nrc1279 14732866

[9] Maman S Witz IP. A History of Exploring Cancer in Context. Nat Rev Cancer (2018) 18(6):359–76. doi: 10.1038/s41568-018-0006-7 29700396

[10] Portela A Esteller M. Epigenetic Modifications and Human Disease. Nat Biotechnol (2010) 28(10):1057–68. doi: 10.1038/nbt.1685 20944598

[11] Wilson AS Power BE Molloy PL. DNA Hypomethylation and Human Diseases. Biochim Biophys Acta (2007) 1775(1):138–62. doi: 10.1016/j.bbcan.2006.08.007 17045745

[12] Esteller M. Epigenetic Gene Silencing in Cancer: The DNA Hypermethylome. Hum Mol Genet (2007) 16 Spec No 1:R50–9. doi: 10.1093/hmg/ddm018 17613547

[13] Xu RH Wei W Krawczyk M Wang W Luo H Flagg K. Circulating Tumour DNA Methylation Markers for Diagnosis and Prognosis of Hepatocellular Carcinoma. Nat Mater (2017) 16(11):1155–61. doi: 10.1038/nmat4997 29035356

[14] Azad N Zahnow CA Rudin CM Baylin SB. The Future of Epigenetic Therapy in Solid Tumours–Lessons From the Past. Nat Rev Clin Oncol (2013) 10(5):256–66. doi: 10.1038/nrclinonc.2013.42 PMC3730253 23546521

[15] Ahuja N Easwaran H Baylin SB. Harnessing the Potential of Epigenetic Therapy to Target Solid Tumors. J Clin Invest (2014) 124(1):56–63. doi: 10.1172/JCI69736 24382390 PMC3871229

[16] Oshiumi H Matsumoto M Hatakeyama S Seya T. Riplet/RNF135, a RING Finger Protein, Ubiquitinates RIG-I to Promote Interferon-Beta Induction During the Early Phase of Viral Infection. J Biol Chem (2009) 284(2):807–17. doi: 10.1074/jbc.M804259200 19017631

[17] Liu Y Wang F Liu Y Yao Y Lv X Dong B. RNF135, RING Finger Protein, Promotes the Proliferation of Human Glioblastoma Cells *In Vivo* and *In Vitro via* the ERK Pathway. Sci Rep (2016) 6:20642. doi: 10.1038/srep20642 26856755 PMC4746631

[18] Jin J Zhao L Li Z. The E3 Ubiquitin Ligase RNF135 Regulates the Tumorigenesis Activity of Tongue Cancer SCC25 Cells. Cancer Med (2016) 5(11):3140–6. doi: 10.1002/cam4.832 PMC5119969 27709798

[19] Yoshihara K Shahmoradgoli M Martinez E Vegesna R Kim H Torres-Garcia W. Inferring Tumour Purity and Stromal and Immune Cell Admixture From Expression Data. Nat Commun (2013) 4:2612. doi: 10.1038/ncomms3612 24113773 PMC3826632

[20] Moh MC Shen S. The Roles of Cell Adhesion Molecules in Tumor Suppression and Cell Migration: A New Paradox. Cell Adhesion Migration (2009) 3(4):334–6. doi: 10.4161/cam.3.4.9246 PMC2802741 19949308

[21] Marcuzzi E Angioni R Molon B Calì B. Chemokines and Chemokine Receptors: Orchestrating Tumor Metastasization. Int J Mol Sci (2018) 20(1): 96. doi: 10.3390/ijms20010096 PMC6337330 30591657

[22] Galuppini F Dal Pozzo CA Deckert J Loupakis F Fassan M Baffa R. Tumor Mutation Burden: From Comprehensive Mutational Screening to the Clinic. Cancer Cell Int (2019) 19:209. doi: 10.1186/s12935-019-0929-4 31406485 PMC6686509

[23] Hendriks LE Rouleau E Besse B. Clinical Utility of Tumor Mutational Burden in Patients With non-Small Cell Lung Cancer Treated With Immunotherapy. Trans Lung Cancer Res (2018) 7(6):647–60. doi: 10.21037/tlcr.2018.09.22 PMC6249615 30505709

[24] Li X Cheung KF Ma X Tian L Zhao J Go MYY. Epigenetic Inactivation of Paired Box Gene 5, a Novel Tumor Suppressor Gene, Through Direct Upregulation of P53 is Associated With Prognosis in Gastric Cancer Patients. Oncogene (2011) 31(29):3419–30. doi: 10.1038/onc.2011.511 22105368

[25] Xu L Li X Chu ES Zhao G Go MY Tao Q. Epigenetic Inactivation of BCL6B, a Novel Functional Tumour Suppressor for Gastric Cancer, is Associated With Poor Survival. Gut (2012) 61(7):977–85. doi: 10.1136/gutjnl-2011-300411 21917650

[26] Sousa B Pereira J Paredes J. The Crosstalk Between Cell Adhesion and Cancer Metabolism. Int J Mol Sci (2019) 20(8):1933. doi: 10.3390/ijms20081933 PMC6515343 31010154

[27] Balkwill FR. The Chemokine System and Cancer. J Pathol (2012) 226(2):148–57. doi: 10.1002/path.3029 21989643

[28] Cadena C Ahmad S Xavier A Willemsen J Park S Park JW. Ubiquitin-Dependent and -Independent Roles of E3 Ligase RIPLET in Innate Immunity. Cell (2019) 177(5):1187–200 e16. doi: 10.1016/j.cell.2019.03.017 31006531 PMC6525047

[29] Tu T Buhler S Bartenschlager R. Chronic Viral Hepatitis and its Association With Liver Cancer. Biol Chem (2017) 398(8):817–37. doi: 10.1515/hsz-2017-0118 28455951

[30] Chiappinelli KB Strissel PL Desrichard A Li H Henke C Akman B. Inhibiting DNA Methylation Causes an Interferon Response in Cancer *via* dsRNA Including Endogenous Retroviruses. Cell (2015) 162(5):974–86. doi: 10.1016/j.cell.2015.07.011 PMC4556003 26317466

[31] Roulois D Loo Yau H Singhania R Wang Y Danesh A Shen SY. DNA-Demethylating Agents Target Colorectal Cancer Cells by Inducing Viral Mimicry by Endogenous Transcripts. Cell (2015) 162(5):961–73. doi: 10.1016/j.cell.2015.07.056 PMC4843502 26317465

[32] Liu J Liu Y Meng L Liu K Ji B. Targeting the PD-L1/DNMT1 Axis in Acquired Resistance to Sorafenib in Human Hepatocellular Carcinoma. Oncol Rep (2017) 38(2):899–907. doi: 10.3892/or.2017.5722 28627705 PMC5561980

[33] Yang H Bueso-Ramos C DiNardo C Estecio MR Davanlou M Geng QR. Expression of PD-L1, PD-L2, PD-1 and CTLA4 in Myelodysplastic Syndromes is Enhanced by Treatment With Hypomethylating Agents. Leukemia (2014) 28(6):1280–8. doi: 10.1038/leu.2013.355 PMC4032802 24270737

[34] Galon J Bruni D. Approaches to Treat Immune Hot, Altered and Cold Tumours With Combination Immunotherapies. Nat Rev Drug Discovery (2019) 18(3):197–218. doi: 10.1038/s41573-018-0007-y 30610226

[35] Chan TA Yarchoan M Jaffee E Swanton C Quezada SA Stenzinger A. Development of Tumor Mutation Burden as an Immunotherapy Biomarker: Utility for the Oncology Clinic. Ann Oncol (2019) 30(1):44–56. doi: 10.1093/annonc/mdy495 30395155 PMC6336005

