# Supplementary material for: RNF135 Promoter Methylation Is Associated With Immune Infiltration and Prognosis in Hepatocellular Carcinoma
Source: Front Oncol. 2022 Jan 25;11:752511. doi: 10.3389/fonc.2021.752511 (PMC8821516; doi:10.3389/fonc.2021.752511)
Supplement: Supplementary file 1 [file DataSheet_1.docx]

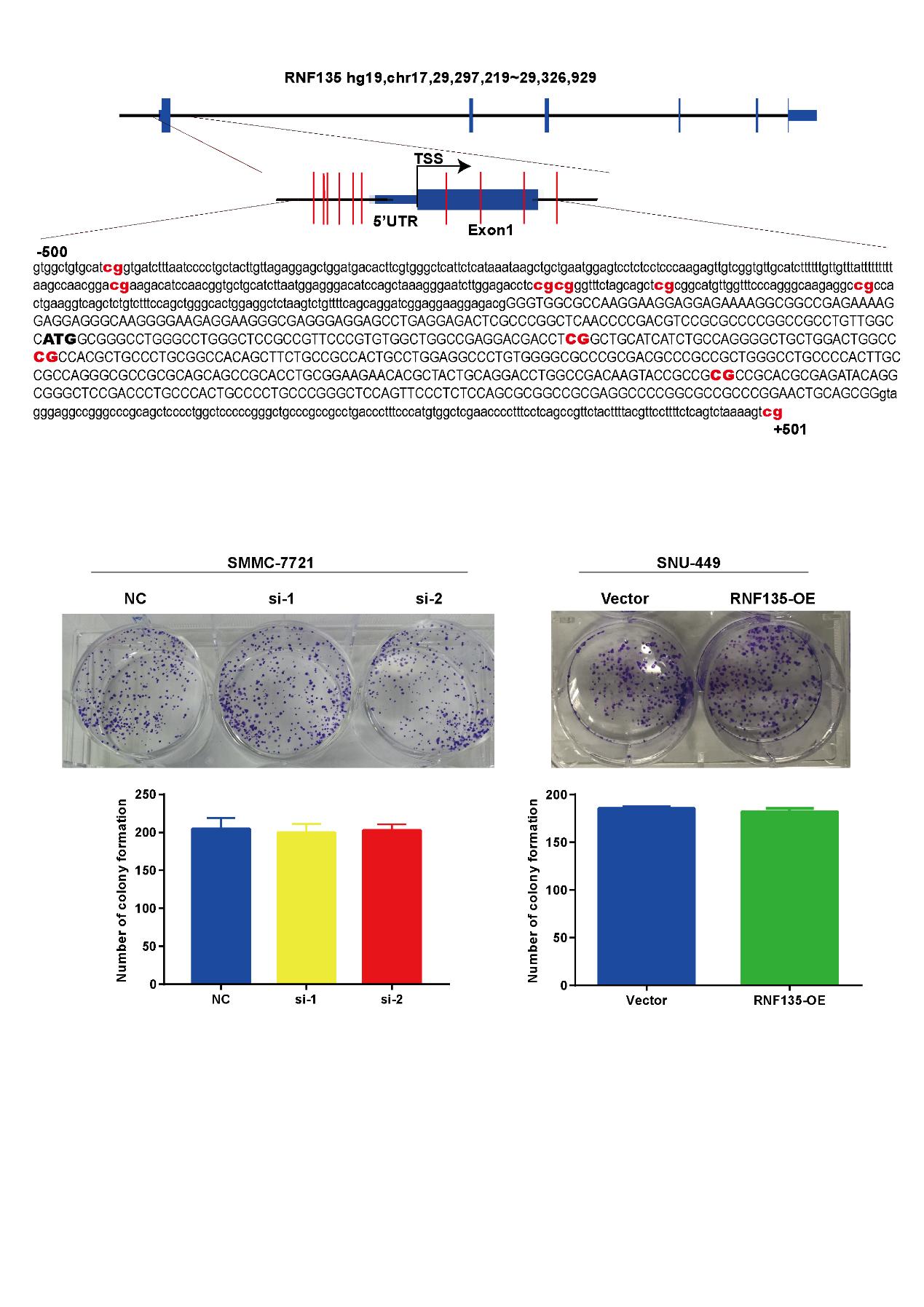


**Supplementary Figure 1. The distribution of 10 CpG sites around transcription start sites**

10 CpG sites around transcription start sites was defined as promoter region. The methylation level of RNF135 promoter was evaluated by the average methylation level of 10 CpG sites.


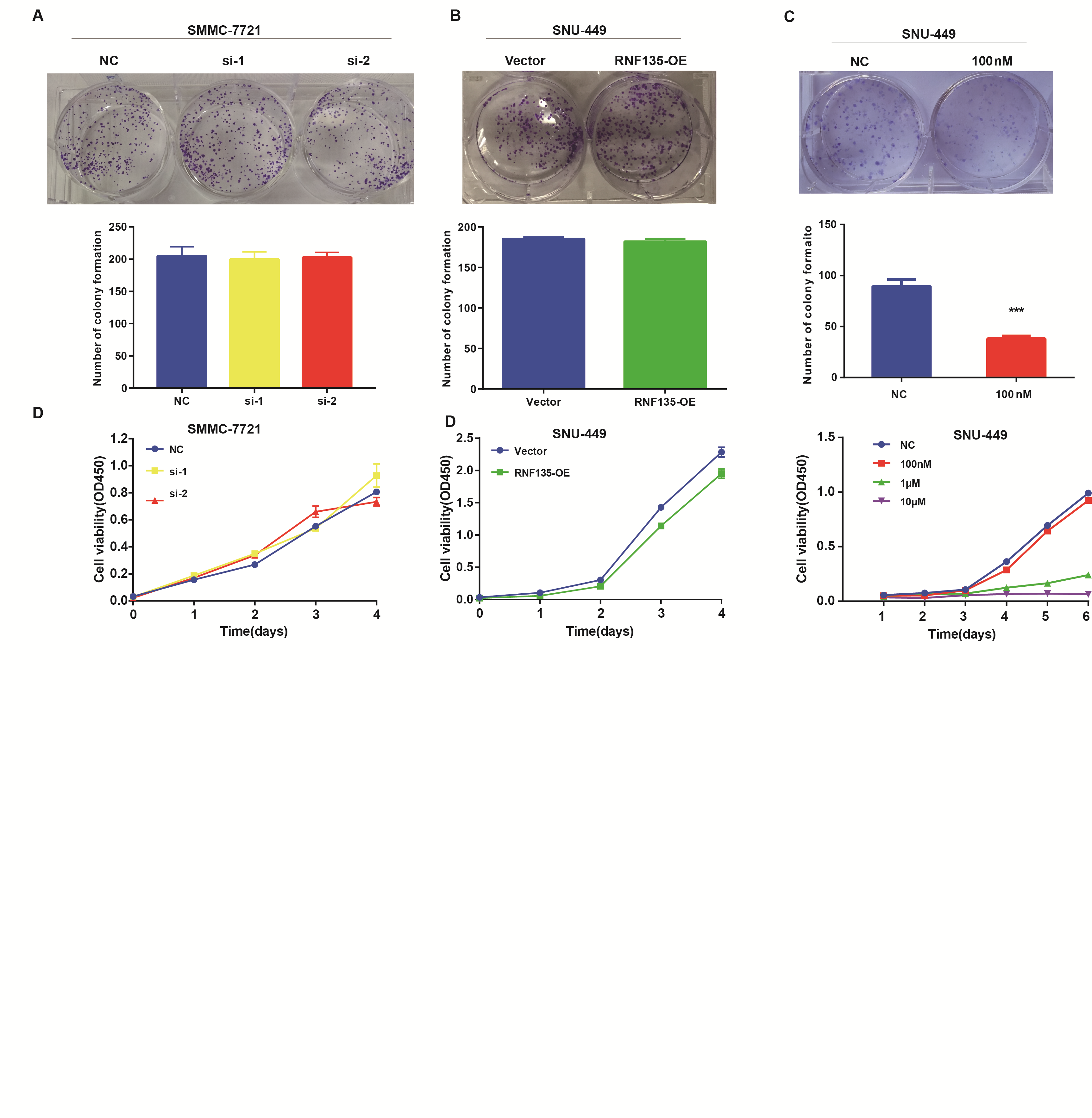


**Supplementary Figure 2. The effects of RNF135 on** **colony formation and cell proliferation**

(A, B, C) The colony formation assay was used to detect colony formation ability (up), and student's t-test for unpaired was applied between experimental group and control respectively (down). The colony formation assay showed that RNF135 has no apparent effect on colony formation. Decitabine inhibited cell colony formation. (D) Cell ability assay was used to evaluate the cell proliferation ability. There was no significant difference between experimental and control group. And 1μM and 10μM decitabine inhibited cell proliferation. All experiments were repeated three times, and data were expressed as mean ±SD.

Table S1. Distribution of patient characteristics by survival status

|  | **Alive (n=244)** | | **Dead (n=121)** | |  |
| --- | --- | --- | --- | --- | --- |
| **Variable** | ***n*** | **%** | ***n*** | **%** | ***P* value** |
| **Sex** |  |  |  |  |  |
| Male | 169 | 68.70% | 77 | 31.30% | 0.28 |
| Female | 75 | 63.00% | 44 | 37.00% |  |
| **Age** |  |  |  |  |  |
| <60 | 121 | 69.90% | 52 | 30.10% | 0.233 |
| >=60 | 123 | 64.10% | 69 | 35.90% |  |
| **Family history of cancer** |  |  |  |  |  |
| No | 163 | 70.60% | 68 | 29.40% | **0.038** |
| Yes | 74 | 59.70% | 50 | 40.30% |  |
| **HBV/HCV infection** |  |  |  |  | **0.003** |
| No | 125 | 60.70% | 81 | 39.30% |  |
| Yes | 118 | 75.60% | 38 | 24.40% |  |
| **Alcohol consumption** |  |  |  |  |  |
| No | 158 | 65.60% | 83 | 34.40% | 0.37 |
| Yes | 85 | 70.20% | 36 | 29.80% |  |
| **Tumor stage** |  |  |  |  |  |
| Ⅰ&Ⅱ | 200 | 73.50% | 72 | 26.50% | **<0.001** |
| Ⅲ&Ⅳ | 43 | 47.30% | 48 | 52.70% |  |
| **Vascular invasion** |  |  |  |  |  |
| No | 164 | 68.60% | 75 | 31.40% | 0.524 |
| Yes | 79 | 65.30% | 42 | 34.70% |  |
| **Recurrence/Progression** |  |  |  |  |  |
| No | 98 | 66.20% | 50 | 33.80% | 0.417 |
| Yes | 117 | 70.50% | 49 | 29.50% |  |
| **Expression** |  |  |  |  |  |
| Low | 102 | 62.20% | 62 | 37.80% | 0.088 |
| High | 142 | 70.60% | 59 | 29.40% |  |
| **Methylation** |  |  |  |  |  |
| Low | 177 | 72.50% | 67 | 27.50% | **0.001** |
| High | 67 | 55.40% | 54 | 44.60% |  |

Bold values represent significant differences.

Table S2. Univariate Cox regression analysis of potential prognostic factors for HCC patients

| **Variable** | **HR (95% CI)** | ***P* value** |
| --- | --- | --- |
| **Sex** |  |  |
| Male | 1.000 | 0.507 |
| Female | 1.134 (0.782~1.643) |  |
| **Age** |  |  |
| <60 | 1.000 | 0.313 |
| >=60 | 1.204 (0.839~1.726) |  |
| **Family history of cancer** |  |  |
| No | 1.000 | 0.248 |
| Yes | 1.24 (0.861~1.778) |  |
| **HBV/HCV infection** |  |  |
| No | 1.000 | **0.002** |
| Yes | 0.541 (0.368~0.795) |  |
| **Alcohol consumption** |  |  |
| No | 1.000 | 0.714 |
| Yes | 0.929 (0.628~1.375) |  |
| **Tumor stage** |  |  |
| Ⅰ&Ⅱ | 1.000 | **<0.001** |
| Ⅲ＆Ⅳ | 2.375 (1.646~3.426) |  |
| **Vascular invasion** |  |  |
| No | 1.000 | 0.246 |
| Yes | 1.251 (0.857~1.827) |  |
| **Recurrence/Progression** |  |  |
| No | 1.000 | 0.133 |
| Yes | 1.333 (0.903~1.969) |  |
| **Expression** |  |  |
| Low | 1.000 | 0.056 |
| High | 0.706 (0.494~1.008) |  |
| **Methylation** |  |  |
| Low | 1.000 | **<0.001** |
| High | 1.960 (1.367~2.810) |  |

Bold values represent significant differences.
